# Identification of genetic defects underlying FVII deficiency in 10 patients belonging to eight unrelated families of the North provinces from Tunisia

**DOI:** 10.1186/1746-1596-7-92

**Published:** 2012-08-08

**Authors:** Hejer Elmahmoudi, Fatma Ben-lakhal, Wijden Elborji, Asma Jlizi, Kaouther Zahra, Rim Sassi, Moez Zorgan, Balkis Meddeb, Amel Elgaaied Ben Ammar, Emna Gouider

**Affiliations:** 1 Laboratory of Genetics, Immunology and Human Pathologies, Tunis, Tunisia; 2 Hemophilia Treatment Center, Aziza Othmana Hospital, Tunis, Tunisia

**Keywords:** FVII deficiency, *F7* gene, Mutations, Polymorphisms, Tunisia

## Abstract

Inherited factor VII (FVII) deficiency is a rare disorder characterized by a bleeding phenotype varying from mild to severe. To date, more than 200 mutations have been described along the *F7* gene encoding for FVII. The aim of this study was the identification of genetic defects underlying FVII deficiency in 10 patients belonging to eight unrelated families of the North provinces from Tunisia. Mutation detection was performed by sequencing the whole *F7* gene coding region, exon-intron boundaries and about 400 bp of the promoter region. We identified 5 mutations in five unrelated families; the novel p.F328Y mutation and the reported mutations: p.R304Q, p.M298I, IVS1aG > A and p.G-39G. For the remaining 5 patients we didn’t identified any mutations using PCR/Sequencing protocol. In conclusion, this study represents the first comprehensive molecular series of FVII deficiency affected patients in Tunisia from the North. We will try in the future to continue the molecular study for Tunisian patients from Center and South provinces in order to have a complete idea about the FVII deficiency mutational profile in our country.

**Virtual slides:**

The virtual slide(s) for this article can be found here: http://www.diagnosticpathology.diagnomx.eu/vs/1288044089753085

## Introduction

Factor VII deficiency is an inherited rare bleeding disorder, which results from decreased or absence of coagulation FVII. Factor VII deficiency is transmitted according the autosomic way and affects 1/500000 population [1]. The clinical features are heterogeneous, ranging from severe life-threatening hemorrhages, such as cerebral, gastrointestinal, and joint hemorrhages, to miscellaneous minor bleeding. The severity isn’t correlated with FVII activities residual levels. FVII is a serine protease which accelerates the prothrombin conversion. *F7* gene which encodes coagulation molecule responsible for blood coagulation was discovered in 1987. It is located on chromosome 13q34 and contained 9 exons. More than 200 mutations were identified in the entire *F7* gene, especially punctual mutations. The genotype-phenotype relationship in FVII deficiency is variable [1,2]. The clinical phenotype in patients is generally more severe in homozygous or compound-heterozygous than heterozygous patients [2]. In Tunisia, according to the World Federation of Hemophilia (WFH) global survey 2009 [3], 15 patients with FVII deficiency are reported. Twelve of them belong to our Hemophilia Treatment Center of Aziza Othaman. The reported data concerning FVII deficiency in Tunisia were interested in the determination of some polymorphisms frequencies or in the study of molecular defect only in Tunisian Jewish patients [4-6]. The main objective of this study has been to identify the molecular defects in the *F7* gene of Tunisian patients and explain the phenotype data of our patients based on their genotypes. In our knowledge this is the first report of molecular defect in Arab Tunisian patients with FVII deficiency.

## Material and methods

### Patients

The studied subjects belonging to Hemophilia Treatment Center of Aziza Othmana, Tunis were recruited between 2010 and 2011. In order to identify the molecular defects of *F7* gene, we included 10 patients (women-6, men-4) from 8 unrelated families with factor VII activities (FVII:C) ranging from 2% to 40%. Their age ranges between 14 and 64 years old. The hemorrhagic symptoms are presented in Table 1.

**Table 1:** Phenotype and genotype data of Tunisian patients with FVII deficiency

**Family**	**Patient**	**Age**	**Symptoms**	**FVII: C**	**Mutation**	**Location**	**Domain**	**Type**	**Genotype**	**Polymorphism**
1	1	1992	Epistaxis, gastrointestinal, dental extraction	23%	p.F328Y*	Exon 8	Cataltytic	Missense	Heterozygous	M1M1, P0/P0, A1/A1,H5
2	2	1996	Epistaxis, dental extraction, gingival, menorrhagia	26%	p.M298I	Exon 8	Cataltytic	Missense	Heterozygous	M1M2, P0/P10, A1/A2,H5
3	3	1992	Asymptomatic	ND	p.R304Q	Exon 8	Cataltytic	Missense	Homozygous	M1M1, P0/P0, A1/A1,H5
4	4	1991	Hemarthroses, gingival, menorrhagia	40%	IVS1a + 5 G > A	Intron 1a	Propeptide	Splicing	Heterozygous	M1M1, P0/P0, A1/A1,H5
5	5	1985	Epistaxis, dental extraction, gingival	ND	p.G-39G	Exon 1b	Propeptide	Splicing	Heterozygous	M1M1, P0/P10, A1/A2,H5
6	6 7	1980 1974	Epistaxis, dental extraction, gingival, Menorrhagia, epistaxis	2.5% 5%	ND					M1M1, P0/P0, A1/A1,H5
7	8 9	1990 1985	Menorrhagia, epistaxis dental extraction, gingival	4% 4%	ND					M1M1, P0/P0, A1/A1,H5
8	10	1963	Menorrhagia, epistaxis	5%	ND					M1M1, P0/P0, A1/A1,H7

*Novel mutation.

Nucleotide numbers are based on the full sequence published by O’hara et al 1987 using the A of the ATG initiator methionine as +1. Numbering of the amino acids is based on Genebank file NM-000131. Methionine is numbered as −60.

Polymorphisms: M1: Arg at position 353, M2: Gln at position 353. P0: no insertion of 10 bp at position-323 in promoter, P10: insertion of 10 bp at position-323 in promoter. A1: nt C at position −122 in promoter, A2: nt T at position −122 in promoter.

ND: not determined.

## Methods

### DNA extraction and amplification

An informed consent was obtained from each patient prior to blood collection. DNA was extracted from white blood cells by standard phenol chloroform extraction method [7]. PCR primers were used for amplification of the whole coding regions, the exon-intron boundaries and the 5’ flanking region containing the promoter of *F7* gene [8]. PCR was carried out in a 25 ml reaction volume in ABI thermal cycle (Perkin-Elmer Applied Biosystems, Foster City, CA, USA).

### Sequencing

Amplified regions were sequenced using ABI Dye Terminator Cycle Sequencing (Perkin-Elmer Applied Biosystems, Foster City, CA, USA) and analyzed using a capillary sequencer Genetic Analyser ABI PRISM310 (Perkin-Elmer Applied Biosystems, Foster City, CA, USA). Results were analyzed using BLAST program ( http://www.ncbi.nlm.nih.gov/blast) against the normal *F7* gene sequence and the mutations were compared with the factor VII mutation database available at http://europium.csc.mrc.ac.uk/.

## Results

### Clinical data

The symptoms observed in our patients are epistaxis, menorrhagia, bruising and metrroragia. The unique episode of hematoma was observed in one woman after trauma. No patients had experienced intracranial hemorrhage (Table 1). Only five patients are treated with prothrombin complex concentrate (PCC).

### Molecular data

Ten fragments corresponding to putative promoter, coding regions and exon-intron boundaries of the *F7* gene were screened for mutation by PCR/sequencing method. A list of potentially pathogenic mutations and polymorphisms of the *F7* gene is shown in Table 1. The position of mutations and polymorphisms are based on the original *F7* gene reported by O’Hara [9].

We identified 5 different mutations in 5 unrelated patients. The mutations are composed of three missense mutations and 2 splicing site mutations. Among them one mutation is novel.

#### Missense mutations (n = 3)

**Figure 1:**
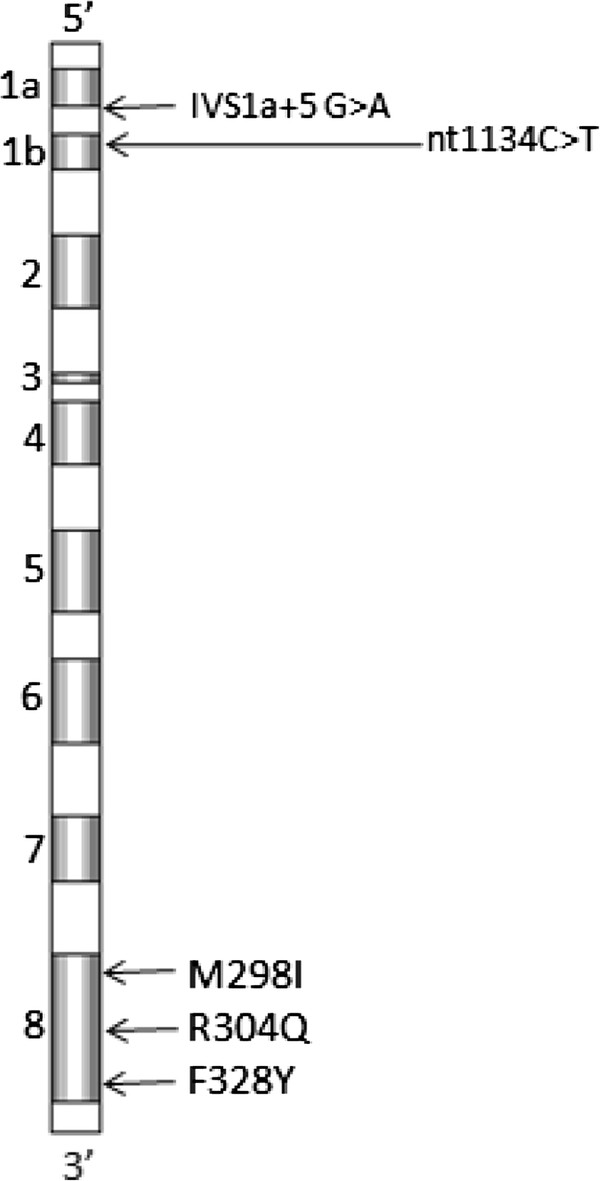
**Localization of identified mutations in 5 unrelated Tunisian patients with FVII deficiency.** The 3 punctual mutations are localized in the catalytic region within exon 8. The 2 splice mutations are localized in intron 1 and in the exon 2. Nucleotide numbers are based on the full sequence published by O’hara et al 1987 using the A of the ATG initiator methionine as +1. Numbering of the amino acids is based on Genebank file NM-000131. Methionine is numbered as −60.

The 3 missense mutations were detected within the *F7* gene (Figure 1) in exon 8; a novel heterozygous mutation p.F328Y (TTC → TAC) in patient 1, a reported heterozygous p.M298I (ATG → ATA) in patient 2 and a reported homozygous G > A transition leading to p.R304Q substitution (CGG → CAG) in patient 3 (Table 1).

#### Splicing site mutations (n = 2)

Sequencing of exon/intron regions using genomic DNA revealed a heterozygous splicing donor site mutation IVS1a + 5 G > A (patient 4) and one silent mutation p.G-39G (GGC > GGT) occurring in the heterozygous state (patient 5). Since the silent mutation is located in + 2 within the exon 1b it is considered as a splice site mutation (Table 1).

#### Polymorphisms (n = 5)

Relating to FVII deficiency, there are various single nucleotide polymorphisms known in *F7* gene, which can lead to an amino acid exchange and have impact on the FVII levels [10]. The investigation of all patients revealed previously described polymorphisms in heterozygous forms concerning 3 patients (patient 2, 5 and 10). The p.R353Q (M1/M2) polymorphism was identified in heterozygous state in patient 2. The 10 bp promoter insertion of nucleotide −323 (P0/P10) and the polymorphic variation -122 T > C (A1/A2) in the promotor region were found in heterozygous state in 2 patients (patient 2 and 5). The repeat polymorphism of intron 7 was identified in one patient (Table 1).

## Discussion

Some substitutions modified directly or indirectly, the binding of factor VII to tissular factor. In our patients we identified 3 missense mutations the novel p.F328Y and the two reported p.R304Q and p.M298I. The novel p.F328Y involves the substitution of a non polar amino acid (F) by a neutral amino acid (Y) in the catalytic domain of the factor VII which may be affects its affinity in binding to the tissular factor. Using PolyPhen, this substitution is predicted to be probably damaging with a score of 1.000 (sensitivity: 0.00; specificity: 1.00). Others studies demonstrate that the p.F328S mutation reduces tissue factor binding, impairs activation by factor Xa and abolishes the coagulant activities [11,12]. Since our novel mutation p.F328Y occurs in the same position as the reported mutation p.F328S, we hypothesis that the p.F328Y may be plays the same role by the reduction of tissue factor binding. The mutation p.F328S is reported in homozygous state and in compound heterozygous state and it is associated with severe phenotype [2]. In our patient the novel mutation p.F328Y is identified in heterozygous state which may be explain the moderate phenotype.

The reported mutations p.M298I and p.R304Q described in the literature, which occur also in the catalytic domain disrupt interactions between factor VII and tissular factor [13,14].

The p.M298I identified in heterozygous state in our patient (patient 2) and also described with the same state [15], is responsible for an inefficient activation of the catalytic site. This mutation is reported in asymptomatic patients when it is in the heterozygous state while in our patient it is associated with epistaxis, dental extraction, gingival and menorrhagia. This may be explained by the presence in heterozygous state of the 3 SNPs; M1M2, P0/P10 >and A1/A2 predicted to reduce the FVII activity [10].

The p.R304Q is reported in homozygous and heterozygous state in many countries indicating that it is a frequent and recurrent mutation [16,17]. In our patient (patient 3) it is associated with homozygous state. Correlation genotype/phenotype for this mutation is in accordance with reported data since this mutation is associated with asymptomatic or paucisymptomatic patients [18], which is the case for our patient who is asymptomatic.

The 2 splicing mutations IVS1a + 5 G > A and the p.G-39G identified in the heterozygous state respectively in patients 4 and 5, are associated with hemarthrosis, gingival, menorrhagia and epistaxis: clinical symptoms associated with heterozygous mutations described above [19,20]. For the patient 5 the presence of the SNPs A1/A2 known to decrease 25% of the FVII levels [21] may also has an effect on the phenotype of our patient.

Among the 5 identified mutations, 3 are localized in the exon 8 (catatytic region) which represent the hot spot region of *F7* gene as reported in literature [10]. Only the p.R304Q occurred within the CpG dinucleotides.

No mutations were found in 5 patients from 3 unrelated families with reduced FVII activities (2.5% to 5%) and they share all the wild alleles in the homozygous state concerning the M1, P0 and A1 SNPs. Only patient number 10 represent 7 repetitions of polymorphism H in exon 7 which may be responsible for the reduced FVII activity. Others data reported also the absence of molecular defect in some patients in their series [17]. The cause may be an intragenic rearrangement escaping our procedure, so we investigate the use of other techniques such as semi-quantitative multiplex PCR or MLPA.

A unique study described molecular pathology within FVII deficiency in Tunisian patients concern Tunisian Jewish [6]. In this study they identified the p.R304Q mutation which is also present in one of our patients despite their different origin, the p.R304Q is a recurrent mutation known in different ethnics groups and populations. The others mutations described in this study are different from our identified mutations which confirm the Arabic origin of our patients.

A few Tunisian studies were interested in the distribution of some polymorphisms (M1/M2 and P0/P10) in patients with FVII deficiency and healthy groups from different Tunisian regions demonstrate that the most frequent alleles are M1/M1 and P0/P0 in all the studied groups and also in groups from the North of Tunisia [4,5]. This distribution is confirmed in our patients who are from the North and in whom we identified only one allele M1/M2 in one patient/7 unrelated patients and we found the allele P0/P10 only in 2 patients. All the others patients are homozygous for the frequent alleles cited above.

## Conclusion

This represents the first comprehensive molecular series of FVII deficiency affected patients in Tunisia. We identified 5 different mutations, one of them is novel. For 5 patients we didn’t identify any mutation with PCR/Sequencing protocol used in this study that’s why we investigate other approaches to identify the molecular defects in these patients. Since our patients are from the North province of Tunisia, we will try in the future to continue the molecular study for Tunisian patients from Center and South provinces in order to have a complete idea about the FVII deficiency mutational profile in our country. This allows us to understand the genotype/phenotype relations of this pathology since it has a heterogeneous clinical manifestation.

## Competing interests

The authors stated that they had no interests which might be perceived as posing a conflict or bias.

## Authors’ contributions

HE performed the research, analyzed the data and wrote the paper; FBL, WE, KZ and MZ contributed in the data collection; AJ and RS performed the research**;** MB, EBAA and GE designed the research. All authors read and approved the final manuscript.
